# Gut microbiota and other factors associated with increased T cell regulation in HIV-exposed uninfected infants

**DOI:** 10.3389/fimmu.2025.1533003

**Published:** 2025-03-03

**Authors:** Michael J. Johnson, Sarah K. Lazarus, Ashlynn E. Bennett, Adriana Tovar-Salazar, Charles E. Robertson, Jennifer M. Kofonow, Shaobing Li, Bruce McCollister, Marta C. Nunes, Shabir A. Madhi, Daniel N. Frank, Adriana Weinberg

**Affiliations:** ^1^ Department of Pediatrics, University of Colorado Anschutz Medical Campus, Aurora, CO, United States; ^2^ Department of Medicine, University of Colorado Anschutz Medical Campus, Aurora, CO, United States; ^3^ Department of Pathology, University of Colorado Anschutz Medical Campus, Aurora, CO, United States; ^4^ South African Medical Research Council Vaccines and Infectious Diseases Analytics Research Unit and Department of Science and Technology/National Research Foundation South African Research Chair Initiative in Vaccine Preventable Diseases, Faculty of Health Sciences, University of the Witwatersrand, Johannesburg, South Africa; ^5^ African Leadership in Vaccinology Expertise, Faculty of Health Sciences, University of the Witwatersrand, Johannesburg, South Africa

**Keywords:** regulatory T cells, gut microbiome, human immunodeficiency virus, HIV-exposed uninfected infants, *Blautia wexleraea*, *Klebsiella pneumoniae*, *Enterobacter cloacae*, *Ruminococcus bromii*

## Abstract

**Introduction:**

Infants exposed to HIV and uninfected (HEUs) are at higher risk of infectious morbidity than HIV-unexposed uninfected infants (HUUs). Multiple immune defects of unknown origin were observed in HEUs. We hypothesized that HEUs have more regulatory and inhibitory checkpoint-expressing T cells (Treg, Tici) than HUUs, which may dampen their immune defenses against pathogens.

**Method:**

We used flow cytometry to measure 25 Treg/Tici subsets in HEUs and HUUs at birth, 6, 28, and 62 weeks of life. We used maternal and infant gut microbiome data reported in a previous study to establish correlations with the Treg/Tici.

**Results:**

At birth, 3 Treg subsets, including the prototypic CD4+FOXP3+ and CD4+FOXP3+CD25+, had higher frequencies in 123 HEUs than in 117 HUUs, and 3 subsets had higher frequencies in HUUs. At 28 and 62 weeks of age, 5 Treg/Tici subsets had higher proportions in HEUs than HUUs. The frequencies of the Treg/Tici subsets that diverged between HEUs and HUUs at birth correlated with differential relative abundances of bacterial taxa in the maternal gut microbiome. The Treg/Tici subsets with significantly different frequencies at subsequent visits correlated with the concurrent composition of the infant gut microbiome. In vitro, treatment of HUU peripheral blood mononuclear cells (PBMC) with bacterial taxa most abundant in HEUs expanded Treg/Tici subsets with higher frequencies in HEUs than HUUs, recapitulating the in vivo correlations. Conversely, in vitro treatment of HEU PBMC did not increase Treg/Tici frequencies. Other factors that correlated with increased Treg/Tici frequencies were low maternal CD4+ T cells in HEUs at birth and male sex in the HUUs at 28 weeks of life.

**Discussion:**

This study shows that maternal and infant gut dysbiosis are central to the increase in Treg/Tici in HEUs and may be targeted by mitigating interventions.

## Introduction

Due to extraordinary advances in the prevention of HIV vertical transmission, 2 million infants exposed to HIV and uninfected (HEUs) are born every year. However, compared with HIV-unexposed infants (HUUs), HEUs have a higher incidence of hospitalization and death due to severe infections during the first 1-2 years of life (1–3). The introduction of universal 3-drug antiretroviral therapy (ART) during pregnancy in 2012 moderately improved the clinical and infectious outcomes of HEUs in Sub-Saharan Africa, but growth and increased hospitalizations in early childhood continued to be reported more than five years after maternal 3-drug ART was implemented, including 20% more stunting at 18 months of age and 2- to 3.5-fold higher rates of hospitalizations due to infections in the first 6 months of age compared with HUUs (4–8). In the US and other regions of the Northern Hemisphere, hospitalizations are increased in HEUs compared to HUUs (9, 10).

HEUs have multiple immunologic dysfunctions that may contribute to their increased risk of severe infection, hospitalization, and death, including increased numbers of regulatory T cells (Treg) (11–24). Due to the broad spectrum of Treg activity, which may dampen T cell, B cell, and antigen presenting cell function, the increase in Treg abundance provides a potential unifying mechanism for the increased susceptibility to severe infections in HEUs. People with HIV also have multiple immunologic abnormalities, including excessive T cell regulation due to high frequencies of Treg and other T cells expressing immunologic checkpoint inhibitors (Tici), which have been associated with accelerated disease progression and high susceptibility to severe infections (25–28). In the general population, Treg/Tici have been associated with decreased immune protection against tumors and viral infections (29–31). We hypothesized that high Treg/Tici frequencies in HEUs may increase their susceptibility to infections in early childhood.

The gut microbiome has emerged as a central element in the education of local and systemic immune responses (32). The human gut harbors 12 to 20% of the total lymphocytes, and most importantly, it is a critical site of innate and adaptive T-cell maturation, second only to the thymus (33, 34). Bacterial taxonomic groups, such as segmented filamentous bacteria, *E. coli*, *B. fragilis* and diverse clostridia (e.g., *Ruminococcaceae* and *Lachnospiraceae*), alter the balance between Treg and conventional T cells (35–38). Studies revealed that bacterial products, such as short-chain fatty acids (SCFAs), tryptophan catabolites, and *B. fragilis*-derived polysaccharide A, promote Treg differentiation and expansion (39–42). The composition of the infant gut microbiome undergoes sequential changes after birth, influenced primarily by the delivery mode, maternal microbiome, and maternal and infant diet (43–51). Previous studies showed differences in the composition of the gut microbiota of people with and without HIV (52–58) and between HEUs and HUUs (59, 60). Moreover, in the same cohort that the current study is based on, we showed significant differences in the gut microbiota of HEUs and HUUs and that infant gut microbiota extensively overlapped with maternal gut microbiota (59).

The Treg hallmark is the transcription factor FOXP3, which inhibits *IFNG* and *IL2* gene transcription and thereby prevents conventional T-cell differentiation (61). Particular importance in the inheritance of Treg characteristics during cell division has been attached to a *FOXP3* intronic regulatory element, conserved non-coding sequences 2 (CNS2), which is completely demethylated in Treg (62). In addition to FOXP3+ Treg, multiple other Treg subsets have been previously validated, including markers shared with Tici (12, 29, 63–65). The goal of this study was to undertake a comprehensive analysis of the relative abundance of Treg/Tici in HEUs and HUUs during the first year of life and to identify factors associated with differences between the two groups, including maternal HIV infection characteristics; infant sex and birth weight; DNA methylation of CNS2 and other loci; and gut microbiome composition.

## Materials and methods

### Study design and approval

The study was approved by the Human Research Ethics Committee at the University of the Witwatersrand (approval number: M171185) and the Colorado Multiple Institutions Review Board (COMIRB 17-0306). Written informed consent was obtained prior to participation in the study. Women with and without HIV were recruited during labor at Chris Hani Baragwanath Academic Hospital in Johannesburg, South Africa. The inclusion criteria for all women were singleton term gestation, planned vaginal delivery, and intent to breastfeed. Women with HIV had to have been prescribed antiretrovirals but not cotrimoxazole during pregnancy. After providing informed consent, maternal and infant metadata were collected from medical records and by interviewing the study participants at 6, 28, and 62 weeks after delivery. Maternal blood was obtained at delivery for CD4+ T-cell and HIV plasma RNA measurements at the local laboratory. Infant cord blood and peripheral blood obtained at 6, 28 and 62 weeks of life were used to measure Treg subsets. Infant rectal swabs obtained at 6, 28, and 62 weeks of life and maternal rectal swabs at delivery were used for microbiome analysis. Mothers and infants who received antibiotic therapy within one month prior to the rectal swab collection, with the exception of cotrimoxazole in HEUs, were excluded from the microbiome analysis.

### Treg/Tici characterization by flow cytometry

PBMC were cryopreserved for viability as previously described and stored at ≤-150°C until use (66, 67). Cryopreserved PBMC/cord blood mononuclear cells were thawed, counted, and processed immediately for phenotypic assessment using two staining panels. Panel A consisted of surface staining with Zombie yellow (viability), CD25 FITC (BioLegend), Lag3 PE (Invitrogen), CTLA4 PE-CF594 (BD Biosciences), CD4 PerCP-Cy5.5 (BD Biosciences), and CD3 Ax700 (BD Biosciences), followed by fixation and permeabilization using the eBioscience Foxp3/Transcription Factor Staining Buffer Set (eBioscience). Intracellular staining was then performed with FoxP3 Ax647 (BD Biosciences), Granzyme B (GranzB) APC-fire750 (BioLegend), IL-10 BV421 (BioLegend) and TGFβ PE-Cy7 (BioLegend). Panel B consisted of surface staining with Zombie yellow (viability), CD4 FITC (BioLegend), CD3 PE-CF594 (BD Biosciences), GITR PerCP-Cy5.5 (BioLegend), TNFR2 PE-Cy7 (BioLegend), CD39 Ax700 (R&D Systems), PD1 APC-Cy7 (BioLegend) and TIGIT BV421 (BioLegend). Intracellular staining consisted of FoxP3 Ax647 (BD Biosciences) and IL-35 PE (BioLegend) antibodies. Analysis was performed using a Galios instrument (Beckman Coulter). The gating strategy is shown in **Supplementary Figure S1**.

*Ex vivo induction of Treg/Tici. Blautia wexlerae* (Cat# BAA-1564, ATCC)*, Lactococcus lactis* (Cat# 19435, ATCC) and *Ruminococcus bromii* (Cat# 27255, ATCC) were subcultured onto Brucella agar plates (Cat# RO1253, Remel) and incubated at 37°C in an anaerobic chamber until colony growth was observed. Clinical isolates of *Klebsiella pneumoniae, Enterobacter cloacae*, and *Proteus mirabilis* from the Microbiology Clinical Laboratory at the University of Colorado Hospital were subcultured onto sheep blood agar plates (Cat# RO 1202, Remel) and incubated overnight at 37°C. A bacterial suspension was generated for each organism by transferring the bacterial colonies to sterile saline and equilibrating them to a 0.5 McFarland standard as measured by a turbidity meter (68). Bacterial suspensions were UV-inactivated for 15 min, aliquoted, and stored frozen at -80°C until use. For stimulation assays, cryopreserved PBMC were thawed, washed, counted with a Guava EasyCyte instrument (Luminex), and resuspended in RPMI 1640 (Corning) supplemented with 10% FBS (Gemini), 2 mM L-glutamine (Gemini), 20 mM HEPES buffer (Corning), and 1% penicillin/streptomycin solution (Gemini) at 10^6^ PBMC/mL. The cells were incubated with bacteria under preoptimized conditions at a multiplicity of infection of 10 colony-forming units per viable PBMC for 7 days at 37°C in a CO_2_ incubator (Thermo Fisher). During the last 16 h of incubation, Brefeldin-A (5 µg/ml; Sigma−Aldrich) was added, after which the cells were washed with PBS (Corning), stained with Zombie Aqua Fixability dye (BioLegend), washed with PBS+1% BSA (Millipore Sigma−Aldrich) and stained for surface markers with GITR BV711, PD1 BV785, Lag3 APC-Cy7 (BioLegend), CD4 PerCP-C75.5, CD25 PECF594, and CD3 Ax700 (BD Biosciences) in BD Horizon Brilliant Stain Buffer Plus (BD Biosciences). The cells were then washed and fixed/permeabilized with the eBioscience FOXP3/Transcription Factor Staining Buffer Set (Invitrogen). The cells were washed with the kit-provided buffer and stained for intracellular markers with IL-10 BV421, CTLA4 BV605, GranzB FITC, FOXP3 PE, TGFβ PE-Cy7 (BioLegend), and IL-35 APC (R&D Systems) in BD Horizon Brilliant Stain Buffer Plus. The cells were then washed and resuspended in PBS+1% paraformaldehyde before acquisition on a NovoCyte Quanteon cytometer (Agilent). PBMC from a study-dedicated leucopack control were used in each run to ensure interassay reproducibility. The data analysis was performed in FlowJo (BD Biosciences). The Treg/Tici% was calculated using live PBMC as the parent. Gating strategy in **Supplementary Figure S3**.

*Analysis of DNA methylation* was performed on CD4+ T cells purified using Miltenyi Biotech CD4 isolation kit (Cat#130-096-533) as per manufacturer’s instructions. The methylation analysis used the Infinium^®^ MethylationEPIC BeadChip (Illumina) as per manufacturer’s instructions.

### Microbiome profiling

The fecal microbiota was profiled by 16S rRNA gene sequencing. Methods and results were previously published (59). All the sequences and corresponding metadata were deposited in the NCBI Sequence Read Archive under BioProject accession number PRJNA816484.

### Statistical analysis

#### T cell subset group comparisons

T cell subsets with frequencies <0.001% at all time points were excluded from the analysis through an *a priori* decision based on the analytical sensitivity of the flow cytometry method. Relative frequencies were used to identify differences in HEUs and HUUs at birth, 6 weeks, 28 weeks, and 62 weeks. These cross-sectional analyses used Wilcoxon rank sum tests from the rstatix package (69), and the FDR was used to correct for multiple comparisons for each visit. Additional analyses examining the effects of covariates such as sex and viral load (<50 or >50 HIV RNA copies/ml of plasma) were also conducted as described above. Spearman’s rank correlation coefficient was used to quantify the strengths of the relationships between continuous variables such as birthweight and CD4 count and the various Treg/Tici subsets. Longitudinal analyses of flow cytometry data used relative frequencies for each cell type and were modeled with a linear mixed effects model (LMM) to account for repeated measurements within each infant. The LMM assumed a normal distribution for the relative frequencies, used a random intercept for infant ID, and included terms for exposure, time (treated as categorical to allow more flexibility) and the interaction between exposure and time. Differences in trends between groups were determined by evaluating F tests for the exposure-by-time interaction. An FDR threshold of <0.1 was used to determine statistical significance for all tests. All analyses were conducted using R version 4.1.3 (70).

#### Methylation analyses

Intensity data (IDAT) files containing the methylation data were read with R and analyzed using the procedure described by Maksimovic et al. (71). Quality control showed that the average detection p values were <0.006 and below the described cutoff. To minimize variation between samples, data normalization was conducted using the *preprocessQuantile* method. Filtering was then used to remove the poor-performing probes. Probes were removed if they failed in one or more samples (n = 5,741), were on sex chromosomes (n = 10,185), were in known SNPs (n = 28,298), or were known to be cross-reactive (n = 24,688), resulting in a final list of 796,947 probes. Differential methylation analysis was subsequently used to identify differences in CpG sites between HEUs and HUUs. M-values were calculated using the lmFit function from the limma package (72), and an FDR cutoff of <0.1 was used to determine significant differences. To interpret the significant CpG sites, gene ontology (GO) analysis was conducted. In addition to identifying differentially methylated CpG sites, differentially methylated regions were also analyzed using the DMRcate package (73).

#### Correlation analyses

Spearman correlation analyses were used to determine the relationships between the microbiome and the Treg/Tici subsets. All significant microbiome data from our previous analysis (59) were compared with the Treg data at 6 weeks, 28 weeks, and 60 weeks. The maternal microbiome was used to correlate with infant Treg data at birth. The Treg data were modeled using HIV exposure status as a confounder, and the residuals of the model were used to evaluate the relationship with the microbiome data. Spearman’s rank correlation coefficient was used to identify the strength of the associations, and an FDR of <0.1 was used to determine statistical significance. All multiple comparison adjustments were performed by visit. Chord diagrams were subsequently constructed for visualization using the Circlize package (74).

#### Ex vivo induction of Treg/Tici

Nonparametric paired comparisons of bacteria-treated and untreated cells were performed using Prism 10.1.1 for MacOS software (GraphPad).

## Results

### Characteristics of the study population

This study enrolled 240 mother–infant pairs from Soweto, including 123 mothers with HIV and 117 without HIV, between June and December 2017. Notable differences between mothers in the two groups were greater chronological age and parity and lower body mass index (BMI) in mothers with HIV (**Table 1**). There were no differences in alcohol or tobacco use or education level between the two groups. Mothers with HIV had a median of 347 CD4+ T cells/µl of blood and <50 HIV RNA copies/ml of plasma.

**Table 1:** Participant characteristics at delivery^#^.

Mothers	Mothers with HIV	Mothers without HIV	p-value
	(N=123)	(N=117)	
**Age (years)**			
Median [Q1, Q3]	30.0 [26.0, 34.5]	25.0 [22.0, 30.0]	< 0.01
**Previous Pregnancies**			
Median [Q1, Q3]	2.00 [1.00, 3.00]	1.00 [0, 2.00]	< 0.01
Missing	2 (1.6%)	5 (4.3%)	
**BMI at 62 Weeks**			
Median [Q1, Q3]	23.9 [20.5, 28.1]	27.1 [21.7, 31.1]	0.02
Missing	51 (41.5%)	59 (50.4%)	
**Smoking During Pregnancy**			
No	114 (92.7%)	113 (96.6%)	0.18
**Alcohol During Pregnancy**			
No	112 (91.1%)	109 (93.2%)	0.55
**CD4+ Cells/µl**			
Median [Q1, Q3]	347 [227, 499]	not applicable	
Missing	7 (5.7%)	not applicable	
**Log HIV RNA copies/ml**			
Median [Q1, Q3]	1.00 [0, 2.06]*	not applicable	
Missing	10 (8.1%)	not applicable	
**Compliant with ART**			
Yes	122 (99.2%)	not applicable	
Infants	HEU	HUU	
	(N=123)	(N=117)	
**Sex**			
Female	65 (52.8%)	56 (47.9%)	0.44
**Mode of Delivery**			
Vaginal	116 (94.3%)	116 (99.1%)	0.07
**Gestational Age (weeks)**			
Mean (SD)	39.1 (2.22)	39.3 (1.52)	0.48
Missing	4 (3.3%)	1 (0.9%)	
**Birth Weight (g)**			
Mean (SD)	3070 (423)	3250 (437)	< 0.01

^#^Maternal BMI was measured at 62 weeks postpartum.

*Target not detected was assigned a numeric of 0 and <20 a value of 10 copies/ml.

ART, antiretroviral treatment; BMI, Body mass index; HIV, Human immunodeficiency virus; HEU, HIV-exposed uninfected; HUU, HIV-unexposed uninfected.

At birth, the HEUs and HUUs had similar gestational ages according to the study design, with an average of 39 weeks. The sex distribution was also similar (**Table 1**). The HEUs had significantly lower birth weights, with a mean of 3070 g, than the 3250 g in HUUs, but no infants met criteria for small for gestational age or large for gestational age. Seven HEUs and one HUU were delivered by emergency C-section for obstetrical indications identified after the initiation of labor.

Infant diet and antibiotic usage, including cotrimoxazole in HEUs, were recorded at each visit (**Supplementary Table S1**). There were no appreciable differences in infant diets between HEUs and HUUs. Mothers and infants who received antibiotics within 1 month prior to stool collection with the exception of cotrimoxazole in HEUs were excluded from the microbiome analyses.

The analysis of the infant gut microbiome at 6, 28, and 62 weeks, maternal gut microbiome at delivery and 62 weeks postpartum, and breastmilk microbiome at 6 weeks postpartum, described in a previous manuscript (59), showed significant differences between HEUs and HUUs and between mothers with and without HIV.

### Treg/Tici subset distribution in HEUs and HUUs

CD4+ and CD8+ Treg/Tici subsets were identified by the expression of previously described Treg/Tici markers (12, 29, 63–65) FOXP3 and/or CD25, CD39, CTLA4, GITR, granzyme B (GranzB), IL10, IL35, LAG3, PD1, TGFβ, TIM3, TIGIT, and/or TNFR2 using two 10-color flow cytometry panels referred to here as panels A and B (gating strategy and fluorescence minus one shown in **Supplementary Figure S1**).

**Figure 1:**
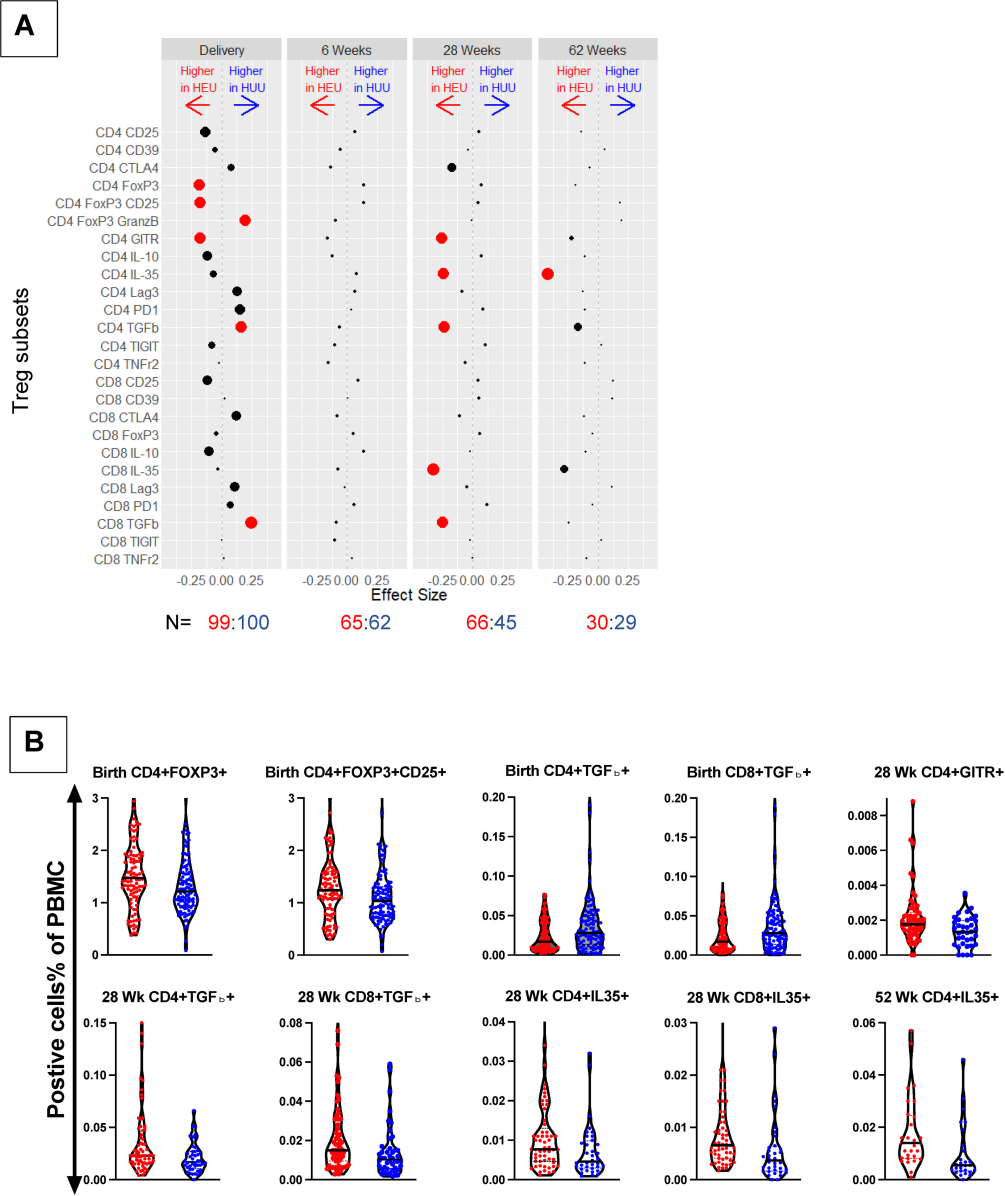
Comparison of Treg/Tici subset frequencies in HEUs and HUUs. Data were derived from a longitudinal cohort of 123 HEUs and 117 HUUs. **(A)** Treg/Tici subsets listed on the y ordinate were compared between HEUs and HUUs using Wilcoxon rank-sum test. The dots represent differences in each Treg/Tici subset at the time points indicated on the graph (top). N (bottom) indicates the number of HEUs in red font and HUUs in blue font that contributed data at each time point. Red dots indicate significant differences with FDR-adjusted p<0.1. The size of each dot is inversely proportional to the unadjusted p value. The distance between each dot and 0 is proportional to the size of the estimated difference. Please see **Supplementary Figure S1** for the gating strategy and **Supplementary Table S2** for a listing of medians and p values. **(B)** Typical examples of the magnitude of differences between HEUs and HUUs Treg/Tici subsets. Graph titles indicate time points and the Treg/Tici subsets. The violin plots show individual data points and medians. HEUs are represented by red dots and HUUs by blue dots.

The comparison of Treg/Tici subsets in cord blood between the two groups (**Figure 1**, **Supplementary Table S2**) revealed significantly greater proportions of CD4+FOXP3+, CD4+FOXP3+CD25+, and CD4+GITR+ Treg in HEUs than HUUs and greater proportions of CD4+FOXP3+GranzB+, CD4+TGFβ+, and CD8+TGFβ+ Treg in HUUs than in HEUs after adjusting the analysis for multiple comparison using the Benjamini-Hochberg false discovery rate (FDR) with p<0.1. Notably, despite higher FOXP3 expression in HEUs, we found an increased frequency of CD4+FOXP3+GranzB+% in HUUs due to much higher expression of GranzB in this group (not depicted). There were no significant differences at 6 weeks of life. At 28 and 62 weeks, the Treg/Tici subsets that significantly differed between the two groups were invariably greater in HEUs than HUUs and included CD4+GITR+, CD4+IL35+, CD4+TGFβ+, CD8+IL35+, and CD8+TGFβ+.

### Effect of maternal HIV disease characteristics on the distribution of Treg/Tici in HEUs

**Figure 2:**
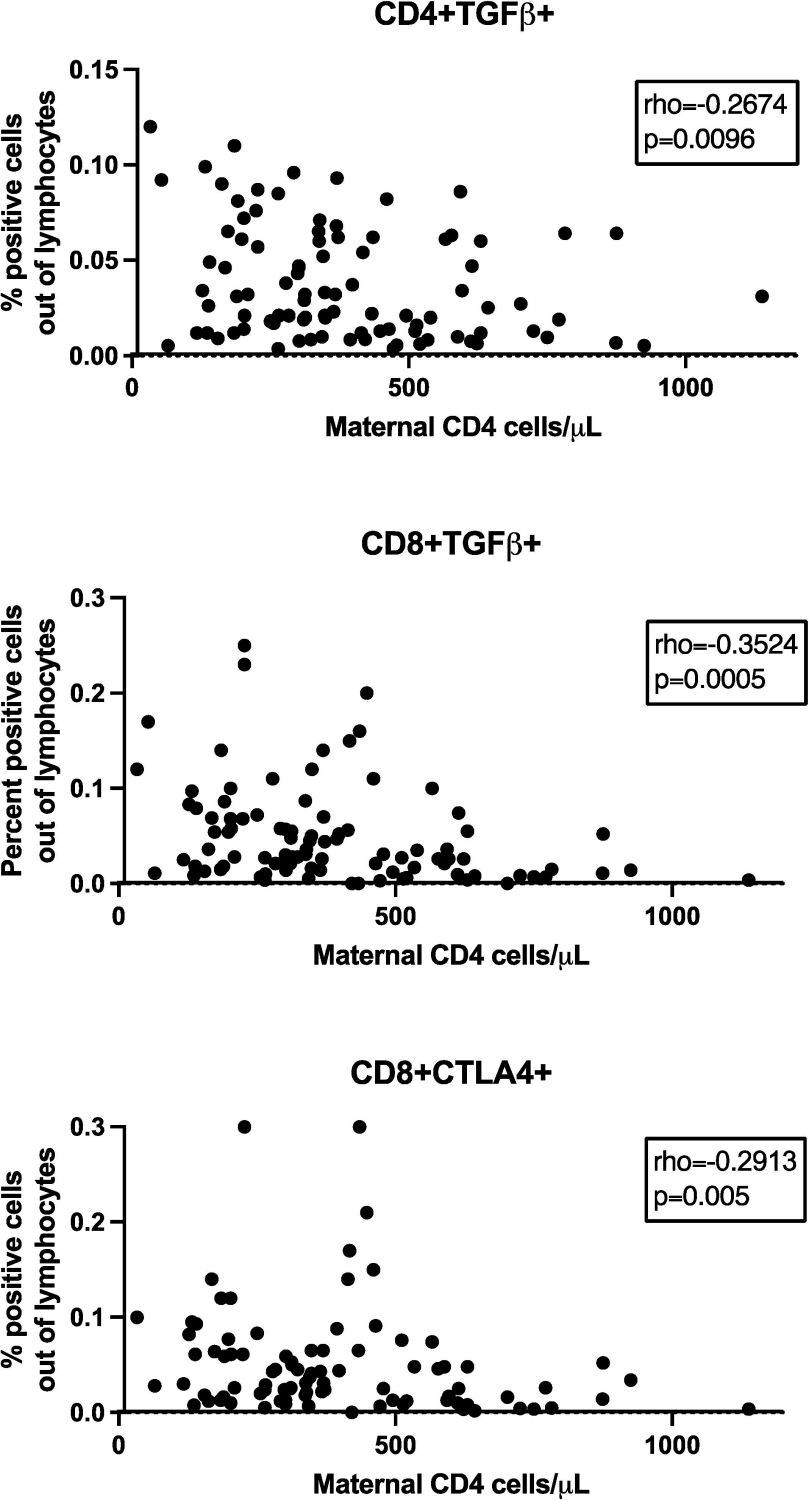
Effect of maternal CD4+ cell numbers on HEU TregTici subsets. Data were derived from 99 HEUs. Graphs show the correlations between maternal CD4+ cell numbers at delivery and frequencies of the Treg/Tici subsets denoted in the title of each graph. The graphs display coefficient of correlations and raw p values calculated by Spearman’s test. FDR p values were ≤0.08.

We investigated the relationship between maternal CD4+ cell numbers and plasma HIV RNA copies/ml at delivery and the frequency of Treg/Tici subsets in HEUs. Spearman correlation analysis of maternal CD4+ cell numbers with all Treg/Tici subsets at all visits revealed significant correlations only at birth and for only three Treg/Tici subsets: CD4+TGFβ+, CD8+TGFβ+ and CD8+CTLA4+ (**Figure 2**). The frequencies of the three Treg subsets increased with decreasing maternal CD4+ cell numbers, with rho values of -0.27 to -0.35, raw p values of 0.0005 to 0.01, and FDR-adjusted p values of 0.03 to 0.08. There were no appreciable differences in the Treg/Tici subset frequencies between HEUs born to mothers with HIV plasma RNA <50 copies/ml or ≥50 copies/ml (not depicted).

### Differential DNA methylation of CD4+ T cells in HEUs and HUUs at birth

We hypothesized that differential Treg distributions between HEUs and HUUs starting at birth might reflect variability in patterns of DNA methylation acquired *in utero*. This hypothesis seemed particularly appropriate for explaining the excess expression of FOXP3 in HEU CD4+ T cells, which has been associated with hypomethylation of several DNA loci (61). However, the analysis of differentially methylated regions in CD4+ T cells from the cord blood of 40 HEUs and 40 HUUs revealed significant differences in a single gene, *thioredoxin-interacting protein* (*TXNIP*), which was hypomethylated in HEUs compared with HUUs (**Supplementary Table S3**). Pathway analyses using the GO and KEGG databases did not reveal any significant differences.

### Effect of infant sex and birth weight on the frequency of Treg/Tici

**Figure 3:**
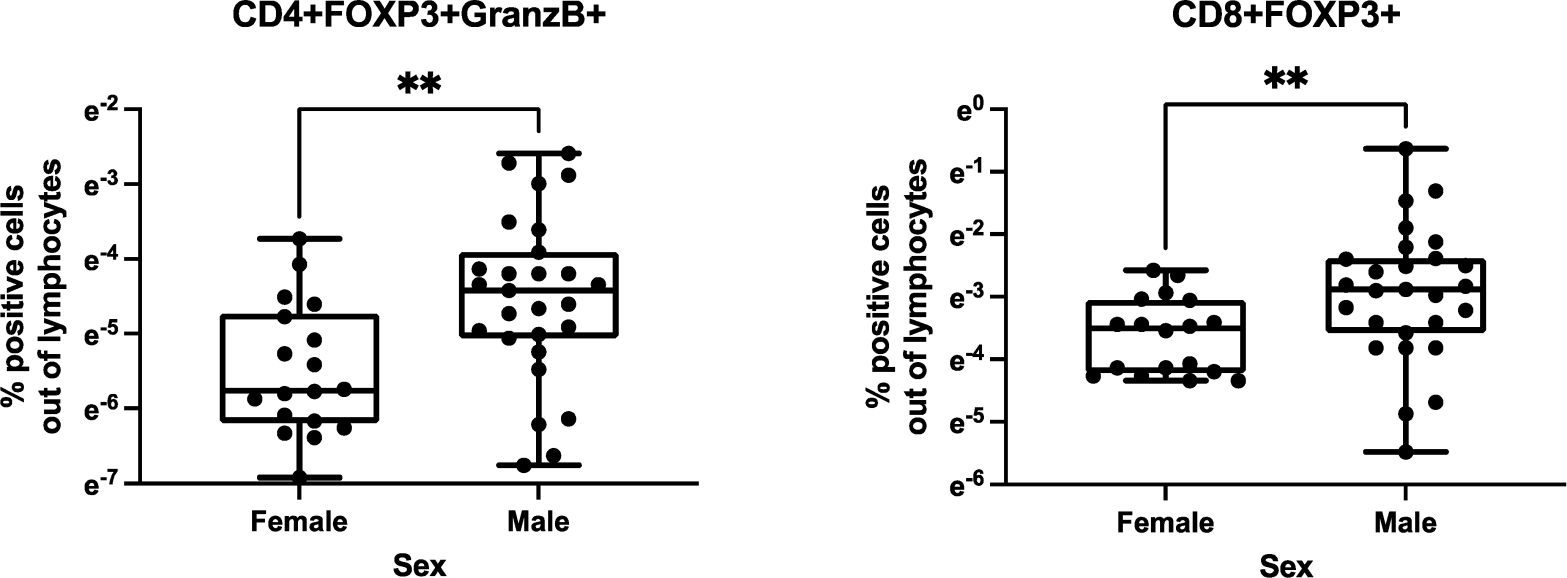
Effect of sex on Treg/Tici distribution in infancy. Data were analyzed in 123 HEUs and 117 HUUs. There was a significant effect of sex only in HUUs at 28 weeks of life. The graphs show the distribution of the Treg/Tici subsets identified in the titles in 18 female and 27 male infants. The asterisks indicate nominal p values<0.01 calculated by Wilcoxon rank-sum test. The FDR adjusted p values were 0.09 for the CD4+FOXp3+GranzB+% comparison and 0.04 for the CD8+FOXP3+% comparison.

We investigated the relationships of birth weight and sex with Treg/Tici distribution. We did not find any relationship between birth weight and Treg/Tici frequencies in HEUs or HUUs (not depicted). We found a significant effect of sex only in HUUs and only at 28 weeks of life, with males showing higher proportions of CD4+FOXP3+GranzB+ and CD8+FOXP3+ Treg (**Figure 3**). Because all infants were born at term according to the study design, we could not evaluate the effect of gestational age.

### Relationship of microbiota with the differential frequencies of Treg/Tici in HEUs and HUUs

**Figure 4:**
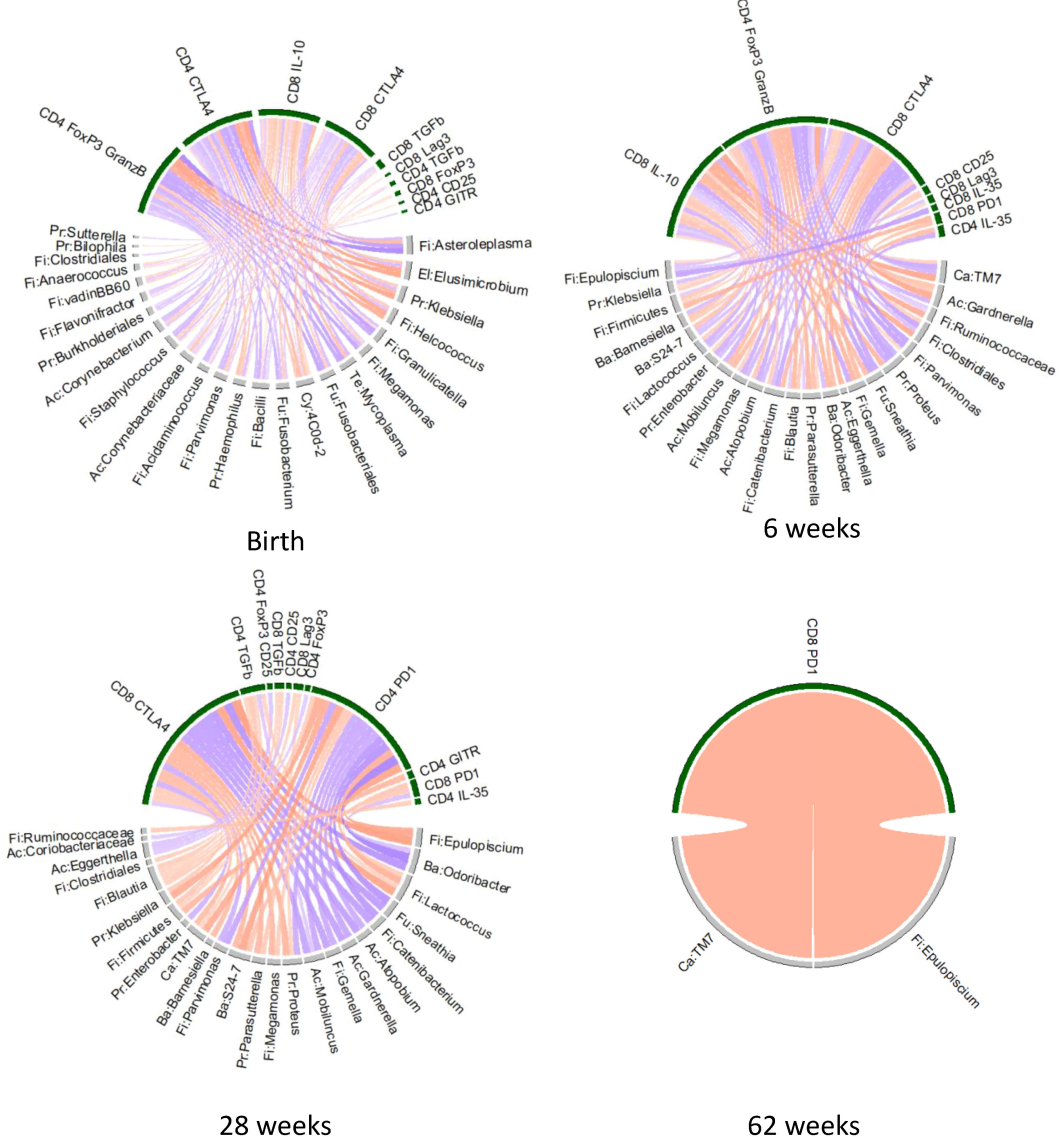
Chord diagram of associations between Treg/Tici subset frequencies and the abundance of gut microbiota that distinguish HEUs from HUUs. Data were derived from HEUs and HUUs with paired Treg/Tici and gut microbiome data at the time points indicated on the graph. The Treg/Tici subsets at birth were correlated with maternal microbiota at delivery. Red chords indicate positive correlations and blue chords negative correlations with FDR p<0.1. Treg/Tici subsets are clustered on the upper part of the circles (green) and bacteria on the lower part (grey). Rho and p values are listed in **Supplementary Table S4**.

We tested the hypothesis that differences in Treg/Tici subsets between HEUs and HUUs could be explained by differences in maternal or infant gut microbiota, which we previously showed to significantly differ between HEUs and HUUs (59) (**Supplementary Figure S2**). To address this hypothesis, we correlated the relative abundances of maternal gut bacterial genera with infant Treg/Tici subsets at birth and the infant microbiota with Treg/Tici subsets at concurrent study visits at 6, 28 and 62 weeks of life. Both analyses focused on bacterial taxa and Treg/Tici subsets that differed between mothers with and without HIV at delivery and/or between HEUs and HUUs. The results revealed multiple significant associations (**Figure 4**, **Supplementary Table S4**). The number of associations decreased over time, due in part to the convergence of the gut microbiota of HEUs and HUUs over time and the reduction in the number of Treg/Tici subsets with differential frequencies. The subsets that correlated with the relative abundance of bacterial taxa in the gut expressed CD25, CTLA4, FOXP3, FOXP3 and GranzB, IL-10, IL-35, Lag3, PD-1, TGFβ, and/or TNFR2. Multiple bacterial taxa correlated with the frequencies of Treg/Tici (**Figure 4**, **Supplementary Table S4**), some of which exhibited significant correlations at multiple time points.

We further postulated that if the relationships between microbial products and Treg/Tici frequencies were causal, they would be reproducible *in vitro*. To test this hypothesis, we identified bacterial taxa with higher abundance in HEUs than HUUs and significant positive correlations with Treg/Tici subsets and classified them as Category A bacteria. Conversely, we classified as Category B bacteria, the taxa with higher abundance in HUUs than HEUs and significant Treg/Tici positive correlations. We reasoned that if our hypothesis was correct, *in vitro* treatment of PBMC from HUUs with Category A bacterial products would increase the frequencies of Treg/Tici generally higher *in vivo* in HEUs than HUUs. Conversely, *in vitro* treatment of HEU PBMC with Category B bacterial products would increase the frequencies of Treg/Tici generally higher *in vivo* in HUUs. The bacterial taxa included in Category A were *Lactococcus*, *Klebsiella*, *Blautia*, and *Ruminococcus. Blautia* is a Firmicute that belongs to the family of *Lachnospiraceae* (75) and an SCFA producer (75). The relative abundance of *Blautia* sp. was associated with increased expression of FOXP3, GranzB, CTLA4, PD1, TGFβ and Lag3 in our study participants. *Lactococcus* is also a Firmicute that can produce acetate and was associated with increased numbers of Treg/Tici expressing PD1, TGFβ, and CTLA4. *Ruminococcus* is a Firmicute that can produce butyrate (76, 77). Its abundance was positively correlated with the expression of IL10 and IL35 in our participants. Bacteria included in Category B were *Enterobacter* and *Proteus*, both belonging to the phylum Proteobacteria, family *Enterobacteriaceae*, which are commonly found in the infant gut microbiome (78). In our study, these bacteria were associated with increased expression of IL10, CTLA4, LAG3, PD1, and TNFr2.

**Figure 5:**
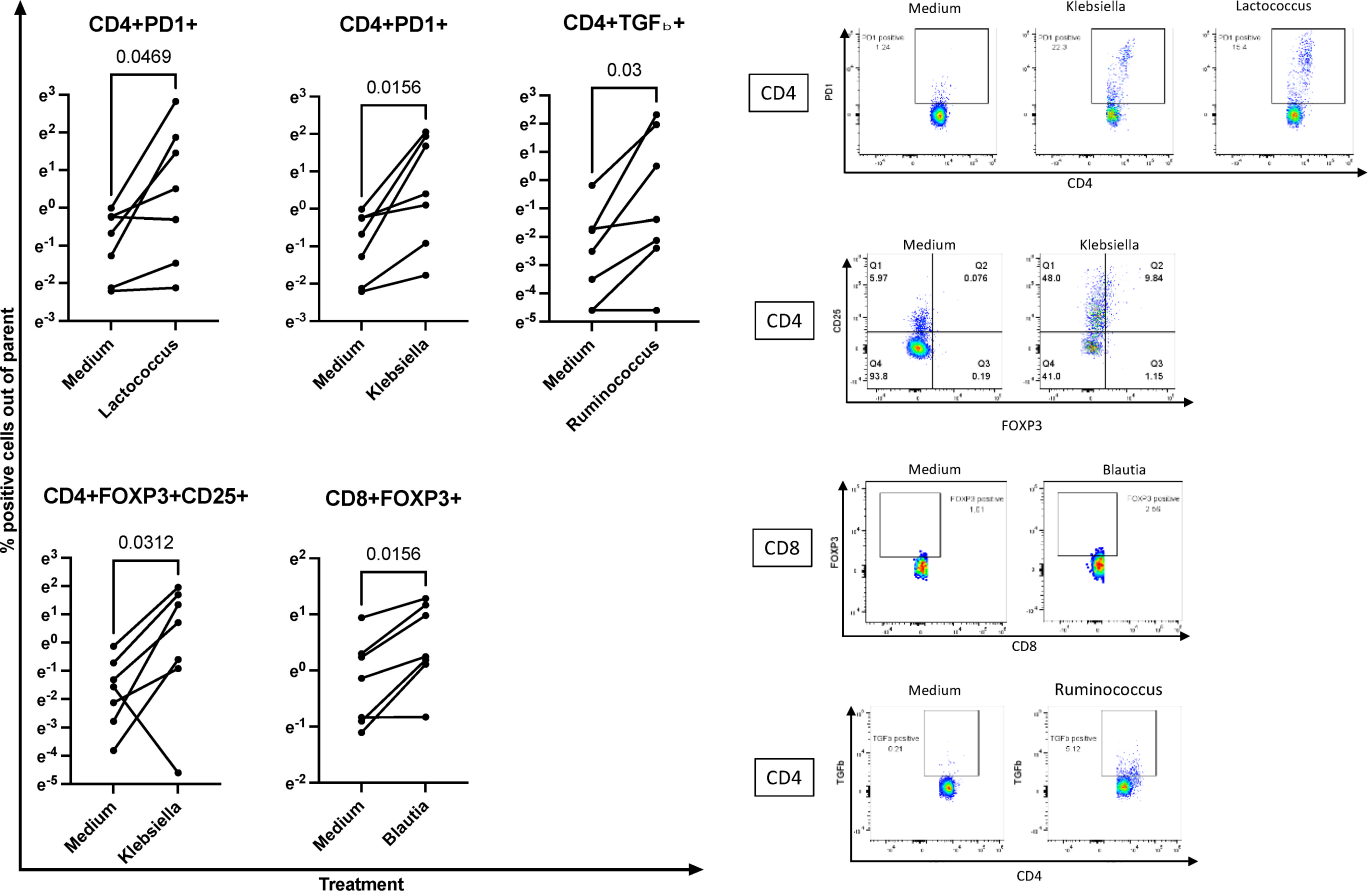
Ex vivo treatment of PBMC with bacterial isolates recapitulates *in vivo* associations with Treg/Tici subsets. Left panels: Data were generated using PBMC from 7 HUUs each treated for 7 days with the UV-inactivated bacterial cultures indicated on each graph. P values were calculated with Wilcoxon matched-pairs signed rank test. Right panels show typical flow cytometric representations of the data summarized in the left panels. Full gating strategies are shown in **Supplementary Figure S3**. Please see **Supplementary Figure S4** for examples of bacterial isolates that did not expand Treg/Tici subsets in HEU PBMC and **Supplementary Figure S5** for effect on adult PBMC.

The *in vitro* experiments were executed in accordance with previous studies that characterized the effect of bacterial products on immune cell subsets (79–83). Using preoptimized conditions, we treated PBMC *in vitro* with the UV-inactivated equivalent of 10 colony-forming units/cell or medium control for 7 days. At the end of the incubation, we measured the frequencies of Treg/Tici expressing FOXP3, CD25, CTLA4, GITR, GranzB, Lag3, IL-10, IL-35, and/or TGFβ (the gating strategy is shown in **Supplementary Figure S3**). PBMC from seven HUUs showed significant increases in the proportions of CD4+PD1+ Treg/Tici when treated with *L. lactis* or *K. pneumoniae* compared to medium control (**Figure 5**). In addition, CD4+FOXP3+CD25+ Treg were significantly increased by *K. pneumoniae*, CD8+FOXP3+ by *B. wexlerae*, and CD4+TGFβ+ by *R. bromii* (**Figure 5**). Notably, these *in vitro* effects largely replicated the correlations observed *in vivo* (**Figure 4**, **Supplementary Table S4**). In contrast, UV-inactivated bacterial treatment did not significantly increase the proportions of any Treg/Tici subsets in HEU PBMC (**Supplementary Figure S4**). The results observed in HUUs were largely replicated in three experiments using healthy donor adult PBMC (**Supplementary Figure S5**).

## Discussion

In this study, we identified differences in the frequencies of Treg/Tici subsets between HEUs and HUUs in the first year of life and established correlations with maternal and infant characteristics. The most prominent factor associated with the frequencies of Treg/Tici was the abundance of certain bacterial taxa in the gut microbiome. We previously identified multiple differences in the HEU and HUU gut microbiota (59) and demonstrate here that these differences are associated with divergent infant Treg/Tici development between these groups.

Notably, differences in the gut microbiota of mothers with and without HIV were associated with differences in Treg/Tici frequencies in HEU and HUU cord blood. These findings are in agreement with previous studies showing that the maternal gut microbiome plays an important role in the development of the infant immune system (84–86). For example, Tanabe et al. showed an association between the maternal gut microbiome and cytokine levels in cord blood (87), and several studies both in humans and in animal models have reported profound effects of the maternal diet on the neonatal immune system mediated by the maternal gut microbiome (84, 88, 89). The communication between the maternal gut microbiome and the fetal immune system is likely to be assisted by bacterial metabolites that freely cross the placenta (87) and deserves further study.

We postulated that some associations between the gut microbiota and Treg/Tici differential frequencies between HEUs and HUUs reflect direct effects of bacterial taxa on the immune system. For four organisms, higher in HEUs than HUUs, including select species of *Blautia, Lactococcus*, *Klebsiella* and *Ruminococcus*, we confirmed their direct relationship with the expansion of Treg/Tici using an *in vitro* model. For two other microorganisms, *Proteus* and *Enterobacter*, which were greater in HUUs than in HEUs, and are commonly found in infant gut microbiomes (78), we could not demonstrate similar relationships. The mechanism underlying the relationships that we identified are likely to involve microbiota-synthesized metabolites, which cross the gut epithelial barrier and inform the immune system development through epigenetic imprinting and post-translational modification of proteins involved in signal transduction (90, 91).

In our previous study, we found that *Blautia* was more abundant in the gut microbiomes of mothers with HIV than in those without HIV, and its relative abundance was positively correlated within mother-infant dyads (59), suggesting that HEUs acquired the bacteria directly from their mothers or through shared local conditions in the gut. In this study, the relative abundance of *Blautia* sp. was associated with increased expression of FOXP3, an effect which was reproduced *in vitro*. *Blautia*’s secondary metabolites and their relationship with human health and disease have raised interest in understanding its physiological properties as well as local gut conditions that modulate its growth (75, 92). Collectively, these observations suggest that *Blautia* may play a role in the immunologic dysfunctions observed in HEUs.

*Lactococcus* produces acetate under low-glucose conditions, which may contribute to T-cell differentiation via the Treg pathway. In fact, *L. lactis* was associated with Treg induction in several animal models (93–95). Less is known about the relationship between *Lactococcus* and Treg in humans. In people with HIV, *Lactococcus* has not stood out in the composition of the gut microbiome but was the second most common microbe identified in the serum of ARV-treated individuals (96). In our study, *Lactococcus* was more abundant in HEUs than in HUUs but not in mothers with HIV compared to those without HIV (59). *In vivo*, *Lactococcus* was associated with increased abundance of Treg/Tici expressing PD1, which was corroborated *in vitro*.

*Ruminococcus* spp. are butyrate producers and, therefore, have the ability to stimulate Treg differentiation (76, 77). There is conflicting information regarding the abundance of *Ruminococcus* in people with HIV (97–100). In our previous study, we did not find differences in the abundance of *Ruminococcus* in mothers with or without HIV. Nevertheless, *Ruminococcus* had a greater relative abundance in HEUs than in HUUs and was correlated with the frequency of CD4+ and CD8+ Treg expressing IL10 or IL35. *In vitro*, *R. bromii* expanded CD4+TGFβ+ Treg. Its role in the immune dysregulation of HEUs deserves to be further elucidated.

*Klebsiella spp* were shown to potentially contribute to the enhanced inflammatory profile of people with HIV and to their neurocognitive impairment (101–103). In our study, *Klebsiella* was positively associated with increased of PD1+ both *in vivo* and *in vitro*. PD1 is an ici that is commonly expressed on Treg and on activated conventional T cells. When bound to its ligands, PDL1 and PDL2, the coupled receptors generate inhibitory intracellular signals that depress the immune response (104). Collectively, these observations suggest that the high abundance of *Klebsiella* in the HEU gut microbiome may contribute to immunologic dysfunction and may warrant studies of interventions to decrease its representation in the gut of HEUs and/or their mothers.

Another factor associated with the excess Treg in HEUs was low maternal CD4+ cell numbers. This association was present at delivery, suggesting that *in utero* communication between mothers and fetuses constituted the underlying mechanism. CD4+ T-cell depletion in people with HIV is largely explained by immune activation in addition to the viral cytopathic effect (105). There is active communication through the placenta between the maternal and fetal immune systems, which may explain the effect of maternal immune activation on fetal immune responses. We have previously shown that, compared with mothers without HIV, mothers with HIV have increased circulating inflammatory marker levels at delivery and that, compared with HUUs, HEUs also have increased plasma inflammatory markers at birth (106). It is conceivable that Treg/Tici expand in HEUs *in utero* to mitigate inflammation and immune activation induced by the mother. This notion is supported by our previous observation that plasma inflammatory marker levels are positively correlated with the frequencies of Treg subsets in pregnant women with HIV (64).

We found two Treg subsets that were significantly greater in HUU males than females at 28 weeks of age. However, we did not find similar differences at other ages in HUUs or in HEUs at any age. Thus, additional confirmatory studies are needed to validate these findings.

We did not find that differential DNA methylation of CD4+ T cells played a role in the difference in Treg variance between HEUs and HUUs. *TXNIP* was the only gene hypomethylated in HEUs. Although *TXNIP* products play a role in hematopoietic cell differentiation, proliferation, apoptosis, and NK cell function (107, 108), a direct contribution to the differentiation of Treg has not been identified to date.

Our study has both limitations and strengths. The number of infants with Treg/Tici measurements decreased from delivery to 62 weeks of life; some of the Treg/Tici may have been in more than one subset because of marker co-expression; and we were unable to investigate potential associations between Treg abundance and ART regimen because all mothers received a fixed dose combination consisting of tenofovir, emtricitabine and efavirenz. Nevertheless, our study has the largest cohort of HEUs and HUUs and the longest follow-up for the comparison of the frequency of Treg/Tici in the two groups and for the association of immunologic and gut microbiome differences between groups. A strength of this study was the *in vitro* verification of microbiome-immune interactions initially identified by *in vivo* associations.

In conclusion, our study established that the frequencies of Treg/Tici subsets differ in HEUs and HUUs from birth to 62 weeks of life, and there is an absolute excess of Treg in HEUs between 28 and 62 weeks of life. We showed in a previous study that a greater proportion of Treg was associated with decreased conventional CD4+ T-cell function in HEUs (12), suggesting that the excess Treg during infancy may underlie the increased susceptibility of HEUs to infections. The factors associated with Treg/Tici development that may be modified through interventions are the infant and maternal gut microbiomes and maternal inflammation. These interventions may result in lower Treg/Tici frequencies in HEUs and potentially lower susceptibility to serious infections compared to the current status quo.

## Data Availability

The datasets presented in this study can be found in online repositories. The names of the repository/repositories and accession number(s) can be found in the article/**Supplementary Material**.
