# Supplementary material for: Gut microbiota and other factors associated with increased T cell regulation in HIV-exposed uninfected infants
Source: Front Immunol. 2025 Mar 3;16:1533003. doi: 10.3389/fimmu.2025.1533003 (PMC11911520; doi:10.3389/fimmu.2025.1533003)
Supplement: Supplementary file 1 [file DataSheet1.docx]

**Table S1 - Feeding pattern and Cotrimoxazole use in HEUs and HUUs**

|  | 6 Weeks  HEU (N = 98) | 6 Weeks HUU (N = 88) | 28 Weeks HEU (N = 86) | 28 Weeks HUU (N = 80) | 62 Weeks HEU (N = 74) | 62 Weeks HUU (N = 74) |
| --- | --- | --- | --- | --- | --- | --- |
| Feeding Method | | | | | | |
| Exclusive Breast Feeding | **83 (84.7%)** | **72 (81.8%)** | **52 (60.5%)** | **40 (50.0%)** | **0 (0%)** | **0 (0%)** |
| Exclusive Formula Feeding | **13 (13.3%)** | **4 (4.5%)** | **19 (22.1%)** | **9 (11.3%)** | **0 (0%)** | **0 (0%)** |
| Mixed* | **2 (2.0%)** | **12 (13.6%)** | **15 (17.4%)** | **31 (38.8%)** | **74 (100%)** | **74 (100%)** |
| **Cotrimoxazole** | | | | | | |
| No | **93 (94.9%)** | **88 (100%)** | **8 (9.3%)** | **79 (98.8%)** | **10 (13.5%)** | **74 (100%)** |
| Yes | **5 (5.1%)** | **0 (0%)** | **77 (89.5%)** | **0 (0%)** | **64 (86.5%)** | **0 (0%)** |
| Missing | **0 (0%)** | **0 (0%)** | **1 (1.2%)** | **1 (1.3%)** | **0 (0%)** | **0 (0%)** |

*Any combination of breastmilk, formula, and/or solids.

A

B

C

**Figure S1. Gating Strategies. Panels A and B** show gating trees used in Treg Panels 1 and 2, respectively.

**Panel C** shows fluorescence minus one (FMO) used to guide the gating of Treg markers

**Table S2. Proportions (%) of Treg subsets in HEUs and HUUs in the first 62 weeks of life**

| Time point | Cell Subset | HEU (median) | HUU (median) | Raw p | FDR p |
| --- | --- | --- | --- | --- | --- |
| Birth | CD4 CD25 | 2.970 | 2.655 | 0.054 | 0.168 |
|  | CD4 CD39 | 0.530 | 0.510 | 0.460 | 0.605 |
|  | CD4 CTLA4 | 0.057 | 0.058 | 0.299 | 0.467 |
|  | *CD4 FoxP3* | *1.450* | *1.190* | *0.009* | *0.069* |
|  | *CD4 FoxP3 CD25* | *1.230* | *1.005* | *0.011* | *0.069* |
|  | *CD4 FoxP3 GranzB* | *0.002* | *0.003* | *0.006* | *0.069* |
|  | *CD4 GITR* | *0.005* | *0.004* | *0.018* | *0.086* |
|  | CD4 IL-10 | 0.010 | 0.009 | 0.107 | 0.223 |
|  | CD4 IL-35 | 0.022 | 0.016 | 0.329 | 0.467 |
|  | CD4 Lag3 | 0.010 | 0.012 | 0.071 | 0.198 |
|  | CD4 PD1 | 0.053 | 0.077 | 0.049 | 0.168 |
|  | *CD4 TGFb* | *0.027* | *0.042* | *0.021* | *0.086* |
|  | CD4 TIGIT | 0.825 | 0.785 | 0.257 | 0.428 |
|  | CD4 TNFR2 | 0.006 | 0.006 | 0.742 | 0.830 |
|  | CD8 CD25 | 0.200 | 0.165 | 0.092 | 0.210 |
|  | CD8 CD39 | 0.056 | 0.058 | 0.764 | 0.830 |
|  | CD8 CTLA4 | 0.031 | 0.047 | 0.084 | 0.210 |
|  | CD8 FoxP3 | 0.010 | 0.010 | 0.534 | 0.668 |
|  | CD8 IL-10 | 0.009 | 0.008 | 0.134 | 0.239 |
|  | CD8 IL-35 | 0.012 | 0.009 | 0.650 | 0.774 |
|  | CD8 Lag3 | 0.037 | 0.051 | 0.119 | 0.229 |
|  | CD8 PD1 | 0.033 | 0.044 | 0.336 | 0.467 |
|  | *CD8 TGFb* | 0.030 | 0.060 | 0.001 | 0.015 |
|  | CD8 TIGIT | 0.400 | 0.425 | 0.999 | 0.999 |
|  | CD8 TNFR2 | 0.002 | 0.002 | 0.828 | 0.863 |
| 6 weeks | CD4 CD25 | 3.990 | 4.155 | 0.456 | 0.770 |
|  | CD4 CD39 | 0.795 | 0.705 | 0.673 | 0.801 |
|  | CD4 CTLA4 | 0.094 | 0.073 | 0.123 | 0.769 |
|  | CD4 FoxP3 | 2.510 | 2.710 | 0.121 | 0.769 |
|  | CD4 FoxP3 CD25 | 2.180 | 2.400 | 0.114 | 0.769 |
|  | CD4 FoxP3 GranzB | 0.006 | 0.006 | 0.282 | 0.770 |
|  | CD4 GITR | 0.004 | 0.003 | 0.217 | 0.770 |
|  | CD4 IL-10 | 0.014 | 0.012 | 0.177 | 0.770 |
|  | CD4 IL-35 | 0.014 | 0.014 | 0.568 | 0.776 |
|  | CD4 Lag3 | 0.083 | 0.100 | 0.493 | 0.770 |
|  | CD4 PD1 | 0.062 | 0.053 | 0.823 | 0.857 |
|  | CD4 TGFb | 0.028 | 0.028 | 0.486 | 0.770 |
|  | CD4 TIGIT | 1.455 | 1.475 | 0.441 | 0.770 |
|  | CD4 TNFR2 | 0.024 | 0.014 | 0.246 | 0.770 |
|  | CD8 CD25 | 0.170 | 0.225 | 0.295 | 0.770 |
|  | CD8 CD39 | 0.105 | 0.130 | 0.993 | 0.993 |
|  | CD8 CTLA4 | 0.009 | 0.009 | 0.348 | 0.770 |
|  | CD8 FoxP3 | 0.028 | 0.033 | 0.574 | 0.776 |
|  | CD8 IL-10 | 0.009 | 0.013 | 0.119 | 0.769 |
|  | CD8 IL-35 | 0.006 | 0.006 | 0.590 | 0.776 |
|  | CD8 Lag3 | 0.150 | 0.160 | 0.787 | 0.855 |
|  | CD8 PD1 | 0.040 | 0.043 | 0.667 | 0.801 |
|  | CD8 TGFb | 0.020 | 0.020 | 0.321 | 0.770 |
|  | CD8 TIGIT | 0.615 | 0.540 | 0.441 | 0.770 |
|  | CD8 TNFR2 | 0.011 | 0.009 | 0.772 | 0.855 |
| 28 weeks | CD4 CD25 | 3.030 | 3.190 | 0.587 | 0.775 |
|  | CD4 CD39 | 0.710 | 0.660 | 0.709 | 0.844 |
|  | CD4 CTLA4 | 0.080 | 0.065 | 0.068 | 0.283 |
|  | CD4 FoxP3 | 2.215 | 2.480 | 0.446 | 0.775 |
|  | CD4 FoxP3 CD25 | 1.765 | 2.060 | 0.620 | 0.775 |
|  | CD4 FoxP3 GranzB | 0.008 | 0.007 | 0.931 | 0.969 |
|  | *CD4 GITR* | *0.002* | *0.001* | *0.015* | *0.093* |
|  | CD4 IL-10 | 0.008 | 0.009 | 0.438 | 0.775 |
|  | *CD4 IL-35* | *0.008* | *0.005* | *0.020* | *0.098* |
|  | CD4 Lag3 | 0.110 | 0.092 | 0.372 | 0.775 |
|  | CD4 PD1 | 0.049 | 0.059 | 0.419 | 0.775 |
|  | *CD4 TGFb* | *0.023* | *0.018* | *0.013* | *0.093* |
|  | CD4 TIGIT | 1.880 | 1.910 | 0.319 | 0.775 |
|  | CD4 TNFr2 | 0.011 | 0.009 | 0.545 | 0.775 |
|  | CD8 CD25 | 0.096 | 0.097 | 0.618 | 0.775 |
|  | CD8 CD39 | 0.155 | 0.160 | 0.601 | 0.775 |
|  | CD8 CTLA4 | 0.007 | 0.005 | 0.250 | 0.775 |
|  | CD8 FoxP3 | 0.034 | 0.040 | 0.560 | 0.775 |
|  | CD8 IL-10 | 0.007 | 0.006 | 0.836 | 0.921 |
|  | *CD8 IL-35* | *0.006* | *0.004* | *0.002* | *0.044* |
|  | CD8 Lag3 | 0.140 | 0.130 | 0.616 | 0.775 |
|  | CD8 PD1 | 0.037 | 0.047 | 0.245 | 0.775 |
|  | *CD8 TGFb* | *0.015* | *0.011* | *0.009* | *0.093* |
|  | CD8 TIGIT | 1.565 | 1.580 | 0.847 | 0.921 |
|  | CD8 TNFR2 | 0.008 | 0.008 | 0.969 | 0.969 |
| 62 weeks | CD4 CD25 | 2.890 | 2.500 | 0.539 | 0.898 |
|  | CD4 CD39 | 0.850 | 0.980 | 0.688 | 0.937 |
|  | CD4 CTLA4 | 0.047 | 0.044 | 0.495 | 0.884 |
|  | CD4 FoxP3 | 1.710 | 1.950 | 0.994 | 0.994 |
|  | CD4 FoxP3 CD25 | 1.170 | 1.320 | 0.934 | 0.973 |
|  | CD4 FoxP3 GranzB | 0.008 | 0.009 | 0.862 | 0.937 |
|  | CD4 GITR | 0.001 | 0.001 | 0.102 | 0.638 |
|  | CD4 IL-10 | 0.010 | 0.008 | 0.339 | 0.858 |
|  | *CD4 IL-35* | *0.014* | *0.006* | *0.002* | *0.041* |
|  | CD4 Lag3 | 0.036 | 0.037 | 0.856 | 0.937 |
|  | CD4 PD1 | 0.057 | 0.042 | 0.408 | 0.858 |
|  | CD4 TGFb | 0.024 | 0.015 | 0.041 | 0.344 |
|  | CD4 TIGIT | 2.880 | 2.920 | 0.850 | 0.937 |
|  | CD4 TNFr2 | 0.006 | 0.005 | 0.412 | 0.858 |
|  | CD8 CD25 | 0.073 | 0.084 | 0.400 | 0.858 |
|  | CD8 CD39 | 0.250 | 0.320 | 0.407 | 0.858 |
|  | CD8 CTLA4 | 0.007 | 0.006 | 0.216 | 0.858 |
|  | CD8 FoxP3 | 0.023 | 0.021 | 0.767 | 0.937 |
|  | CD8 IL-10 | 0.015 | 0.008 | 0.343 | 0.858 |
|  | CD8 IL-35 | 0.008 | 0.006 | 0.038 | 0.344 |
|  | CD8 Lag3 | 0.045 | 0.044 | 0.791 | 0.937 |
|  | CD8 PD1 | 0.041 | 0.028 | 0.724 | 0.937 |
|  | CD8 TGFb | 0.013 | 0.009 | 0.262 | 0.858 |
|  | CD8 TIGIT | 2.650 | 3.090 | 0.857 | 0.937 |
|  | CD8 TNFR2 | 0.005 | 0.004 | 0.476 | 0.884 |

Data were derived from up to 100 participants per group (see also Figure 1). The raw p value was calculated using Wilcoxon rank-sum test. Italics highlight subsets with FDR p<0.1.

**Table S3. Differentially methylated genes in HEU and HUU cord blood CD4+ T cells**

| Gene | Location | Start | End | N cpg | Mean difference (HUU-HEU) | Fisher p value |
| --- | --- | --- | --- | --- | --- | --- |
| ***TXNIP*** | **Chromosome 1** | **145440445** | **145441552** | **3** | **-0.11** | **5.84^-07^** |

A

B

**Figure S2. Differences in maternal and infant gut microbiome**. The graphs show taxa with significantly different abundances between HEUs and HUUs. **Panel A**: Maternal gut microbiome at delivery. **Panel B**: Infant gut microbiomes at the ages indicated on the graphs.

| **Table S4. Correlations between the abundance of infant gut bacterial genera and Treg subsets in HEU and HUU** | | | | | |
| --- | --- | --- | --- | --- | --- |
| Visit | Bacteria | Treg Subset | Spearman Rho | P value | FDR |
| Birth | Firmicutes:Anaerococcus | CD4 CD25 | 0.186221706 | 0.00932815 | 0.07860803 |
|  | Elusimicrobia:Elusimicrobium | CD4 CTLA4 | 0.537077999 | 6.85E-16 | 1.28E-13 |
|  | Firmicutes:Helcococcus | CD4 CTLA4 | 0.516284449 | 1.31E-14 | 1.96E-12 |
|  | Firmicutes:Asteroleplasma | CD4 CTLA4 | -0.495124069 | 2.15E-13 | 2.69E-11 |
|  | Firmicutes:Granulicatella | CD4 CTLA4 | -0.478533952 | 1.70E-12 | 1.59E-10 |
|  | Tenericutes:Mycoplasma | CD4 CTLA4 | -0.463560537 | 9.98E-12 | 6.81E-10 |
|  | Proteobacteria:Klebsiella | CD4 CTLA4 | 0.451337605 | 3.98E-11 | 2.30E-09 |
|  | Fusobacteria:Fusobacteriales | CD4 CTLA4 | -0.429534006 | 4.11E-10 | 2.06E-08 |
|  | Cyanobacteria:4C0d-2 | CD4 CTLA4 | 0.412425591 | 2.30E-09 | 1.01E-07 |
|  | Firmicutes:Bacilli | CD4 CTLA4 | -0.387872658 | 2.30E-08 | 8.62E-07 |
|  | Firmicutes:Parvimonas | CD4 CTLA4 | -0.359736625 | 2.58E-07 | 8.42E-06 |
|  | Firmicutes:Staphylococcus | CD4 CTLA4 | -0.349818585 | 5.74E-07 | 1.48E-05 |
|  | Firmicutes:Megamonas | CD4 CTLA4 | -0.344198076 | 8.92E-07 | 2.23E-05 |
|  | Firmicutes:Acidaminococcus | CD4 CTLA4 | -0.329747478 | 2.66E-06 | 4.87E-05 |
|  | Fusobacteria:Fusobacterium | CD4 CTLA4 | -0.323615192 | 4.17E-06 | 7.11E-05 |
|  | Actinobacteria:Corynebacterium | CD4 CTLA4 | -0.319978797 | 5.41E-06 | 8.78E-05 |
|  | Proteobacteria:Haemophilus | CD4 CTLA4 | 0.315480045 | 7.44E-06 | 0.00011388 |
|  | Proteobacteria:Burkholderiales | CD4 CTLA4 | -0.28234905 | 6.64E-05 | 0.00090589 |
|  | Firmicutes:vadinBB60 | CD4 CTLA4 | 0.272381242 | 0.00012193 | 0.00154993 |
|  | Firmicutes:Flavonifractor | CD4 CTLA4 | -0.264645678 | 0.00019226 | 0.00232574 |
|  | Actinobacteria:Corynebacteriaceae | CD4 CTLA4 | -0.200891932 | 0.00497405 | 0.04441114 |
|  | Firmicutes:Asteroleplasma | CD4 FoxP3 GranzB | -0.594863048 | 5.92E-20 | 4.44E-17 |
|  | Elusimicrobia:Elusimicrobium | CD4 FoxP3 GranzB | 0.544087605 | 2.42E-16 | 9.08E-14 |
|  | Proteobacteria:Klebsiella | CD4 FoxP3 GranzB | 0.537199516 | 6.73E-16 | 1.28E-13 |
|  | Firmicutes:Helcococcus | CD4 FoxP3 GranzB | 0.488690963 | 4.86E-13 | 5.21E-11 |
|  | Firmicutes:Megamonas | CD4 FoxP3 GranzB | -0.463946032 | 9.55E-12 | 6.81E-10 |
|  | Fusobacteria:Fusobacteriales | CD4 FoxP3 GranzB | -0.463553287 | 9.99E-12 | 6.81E-10 |
|  | Tenericutes:Mycoplasma | CD4 FoxP3 GranzB | -0.453212438 | 3.23E-11 | 2.02E-09 |
|  | Firmicutes:Granulicatella | CD4 FoxP3 GranzB | -0.436339139 | 2.02E-10 | 1.08E-08 |
|  | Fusobacteria:Fusobacterium | CD4 FoxP3 GranzB | -0.395303243 | 1.17E-08 | 4.61E-07 |
|  | Proteobacteria:Haemophilus | CD4 FoxP3 GranzB | 0.36996346 | 1.10E-07 | 3.93E-06 |
|  | Firmicutes:Acidaminococcus | CD4 FoxP3 GranzB | -0.357604475 | 3.07E-07 | 9.60E-06 |
|  | Firmicutes:Parvimonas | CD4 FoxP3 GranzB | -0.341489995 | 1.10E-06 | 2.53E-05 |
|  | Firmicutes:Staphylococcus | CD4 FoxP3 GranzB | -0.341303707 | 1.12E-06 | 2.53E-05 |
|  | Firmicutes:Bacilli | CD4 FoxP3 GranzB | -0.338696272 | 1.36E-06 | 2.92E-05 |
|  | Cyanobacteria:4C0d-2 | CD4 FoxP3 GranzB | 0.338049742 | 1.43E-06 | 2.98E-05 |
|  | Actinobacteria:Corynebacterium | CD4 FoxP3 GranzB | -0.32652669 | 3.37E-06 | 6.03E-05 |
|  | Firmicutes:Flavonifractor | CD4 FoxP3 GranzB | -0.276940728 | 9.26E-05 | 0.00121872 |
|  | Proteobacteria:Burkholderiales | CD4 FoxP3 GranzB | -0.227083838 | 0.00145168 | 0.01512162 |
|  | Actinobacteria:Corynebacteriaceae | CD4 FoxP3 GranzB | -0.20599528 | 0.00395649 | 0.03709206 |
|  | Proteobacteria:Sutterella | CD4 GITR | -0.218691631 | 0.00345153 | 0.03276767 |
|  | Firmicutes:Bacilli | CD4 TGFb | -0.210581477 | 0.00320638 | 0.03083062 |
|  | Proteobacteria:Bilophila | CD4 TGFb | 0.185274146 | 0.00970056 | 0.080838 |
|  | Cyanobacteria:4C0d-2 | CD8 CTLA4 | 0.364299426 | 1.77E-07 | 6.04E-06 |
|  | Tenericutes:Mycoplasma | CD8 CTLA4 | -0.355579199 | 3.62E-07 | 1.06E-05 |
|  | Elusimicrobia:Elusimicrobium | CD8 CTLA4 | 0.352125254 | 4.78E-07 | 1.28E-05 |
|  | Proteobacteria:Klebsiella | CD8 CTLA4 | 0.341750791 | 1.08E-06 | 2.53E-05 |
|  | Fusobacteria:Fusobacteriales | CD8 CTLA4 | -0.340698874 | 1.17E-06 | 2.58E-05 |
|  | Firmicutes:Asteroleplasma | CD8 CTLA4 | -0.337395995 | 1.50E-06 | 3.05E-05 |
|  | Firmicutes:Granulicatella | CD8 CTLA4 | -0.336139055 | 1.65E-06 | 3.26E-05 |
|  | Firmicutes:Helcococcus | CD8 CTLA4 | 0.325607786 | 3.61E-06 | 6.29E-05 |
|  | Firmicutes:Bacilli | CD8 CTLA4 | -0.315803728 | 7.27E-06 | 0.00011364 |
|  | Firmicutes:Staphylococcus | CD8 CTLA4 | -0.281380421 | 7.05E-05 | 0.00094476 |
|  | Firmicutes:vadinBB60 | CD8 CTLA4 | 0.273243576 | 0.00011579 | 0.00149733 |
|  | Proteobacteria:Burkholderiales | CD8 CTLA4 | -0.271302246 | 0.00013003 | 0.0016254 |
|  | Actinobacteria:Corynebacterium | CD8 CTLA4 | -0.267673503 | 0.00016113 | 0.00198114 |
|  | Fusobacteria:Fusobacterium | CD8 CTLA4 | -0.260343465 | 0.00024622 | 0.00288545 |
|  | Firmicutes:Megamonas | CD8 CTLA4 | -0.255031266 | 0.00033227 | 0.00377583 |
|  | Firmicutes:Parvimonas | CD8 CTLA4 | -0.251912678 | 0.00039503 | 0.00442201 |
|  | Firmicutes:Acidaminococcus | CD8 CTLA4 | -0.249916305 | 0.0004408 | 0.00486175 |
|  | Proteobacteria:Haemophilus | CD8 CTLA4 | 0.214579427 | 0.00266011 | 0.02660112 |
|  | Actinobacteria:Corynebacteriaceae | CD8 CTLA4 | -0.199477436 | 0.00529487 | 0.04671945 |
|  | Firmicutes:Flavonifractor | CD8 CTLA4 | -0.180450809 | 0.01180735 | 0.09731336 |
|  | Actinobacteria:Corynebacteriaceae | CD8 FoxP3 | 0.193131327 | 0.0069742 | 0.06012242 |
|  | Firmicutes:Staphylococcus | CD8 FoxP3 | 0.191545566 | 0.00746181 | 0.06359495 |
|  | Firmicutes:Asteroleplasma | CD8 IL-10 | 0.424697433 | 6.75E-10 | 3.16E-08 |
|  | Tenericutes:Mycoplasma | CD8 IL-10 | 0.395842059 | 1.11E-08 | 4.61E-07 |
|  | Actinobacteria:Corynebacteriaceae | CD8 IL-10 | 0.355415575 | 3.67E-07 | 1.06E-05 |
|  | Elusimicrobia:Elusimicrobium | CD8 IL-10 | -0.353312086 | 4.34E-07 | 1.21E-05 |
|  | Proteobacteria:Klebsiella | CD8 IL-10 | -0.335001133 | 1.80E-06 | 3.46E-05 |
|  | Proteobacteria:Haemophilus | CD8 IL-10 | -0.331887859 | 2.27E-06 | 4.26E-05 |
|  | Fusobacteria:Fusobacterium | CD8 IL-10 | 0.320494963 | 5.22E-06 | 8.69E-05 |
|  | Firmicutes:Parvimonas | CD8 IL-10 | 0.319747921 | 5.50E-06 | 8.78E-05 |
|  | Actinobacteria:Corynebacterium | CD8 IL-10 | 0.311799166 | 9.62E-06 | 0.00014424 |
|  | Fusobacteria:Fusobacteriales | CD8 IL-10 | 0.310645065 | 1.04E-05 | 0.00015099 |
|  | Firmicutes:Helcococcus | CD8 IL-10 | -0.310569872 | 1.05E-05 | 0.00015099 |
|  | Firmicutes:Megamonas | CD8 IL-10 | 0.30450867 | 1.58E-05 | 0.00022395 |
|  | Firmicutes:Staphylococcus | CD8 IL-10 | 0.299124034 | 2.27E-05 | 0.00031489 |
|  | Firmicutes:vadinBB60 | CD8 IL-10 | -0.264013383 | 0.00019943 | 0.0023742 |
|  | Firmicutes:Anaerococcus | CD8 IL-10 | 0.255863265 | 0.00031717 | 0.00365964 |
|  | Firmicutes:Clostridiales | CD8 IL-10 | -0.23287373 | 0.00108449 | 0.01161958 |
|  | Firmicutes:Granulicatella | CD8 IL-10 | 0.222661057 | 0.00180522 | 0.01854677 |
|  | Cyanobacteria:4C0d-2 | CD8 IL-10 | -0.215717855 | 0.00252081 | 0.02554872 |
|  | Firmicutes:Bacilli | CD8 IL-10 | 0.213744762 | 0.00276664 | 0.0273024 |
|  | Firmicutes:Flavonifractor | CD8 IL-10 | 0.20145584 | 0.00485109 | 0.04393015 |
|  | Firmicutes:Acidaminococcus | CD8 IL-10 | 0.201407117 | 0.0048616 | 0.04393015 |
|  | Cyanobacteria:4C0d-2 | CD8 Lag3 | 0.212039278 | 0.00299643 | 0.02918597 |
|  | Cyanobacteria:4C0d-2 | CD8 TGFb | 0.247555938 | 0.00050123 | 0.00544812 |
|  | Actinobacteria:Corynebacteriaceae | CD8 TGFb | -0.230762262 | 0.00120718 | 0.01275186 |
|  | Firmicutes:Anaerococcus | CD8 TGFb | -0.204259799 | 0.00427927 | 0.03962291 |
|  | Firmicutes:Bacilli | CD8 TGFb | -0.194763564 | 0.00650216 | 0.05670485 |
| Week 6 | Actinobacteria:Atopobium | CD4 FoxP3 GranzB | -0.408385461 | 1.85E-05 | 0.000528393 |
|  | Actinobacteria:Gardnerella | CD4 FoxP3 GranzB | 0.515338857 | 2.55E-08 | 7.64E-06 |
|  | Actinobacteria:Mobiluncus | CD4 FoxP3 GranzB | 0.403088767 | 2.43E-05 | 0.000633016 |
|  | Bacteroidetes:Barnesiella | CD4 FoxP3 GranzB | 0.354736915 | 0.000236393 | 0.004052445 |
|  | Bacteroidetes:Odoribacter | CD4 FoxP3 GranzB | 0.436347202 | 4.08E-06 | 0.000185828 |
|  | Bacteroidetes:S24-7 | CD4 FoxP3 GranzB | -0.307265895 | 0.001593504 | 0.017073259 |
|  | Candidate-division-TM7:Candidate-division-TM7 | CD4 FoxP3 GranzB | -0.378085345 | 8.23E-05 | 0.001543669 |
|  | Firmicutes:Blautia | CD4 FoxP3 GranzB | 0.415973787 | 1.24E-05 | 0.000392557 |
|  | Firmicutes:Catenibacterium | CD4 FoxP3 GranzB | -0.338049097 | 0.000478776 | 0.007030315 |
|  | Firmicutes:Clostridiales | CD4 FoxP3 GranzB | -0.321589656 | 0.000924998 | 0.011326509 |
|  | Firmicutes:Firmicutes | CD4 FoxP3 GranzB | 0.337966521 | 0.000480405 | 0.007030315 |
|  | Firmicutes:Gemella | CD4 FoxP3 GranzB | 0.446028258 | 2.34E-06 | 0.000140265 |
|  | Firmicutes:Lactococcus | CD4 FoxP3 GranzB | -0.381943147 | 6.86E-05 | 0.001328148 |
|  | Firmicutes:Megamonas | CD4 FoxP3 GranzB | -0.40598152 | 2.09E-05 | 0.000570875 |
|  | Firmicutes:Parvimonas | CD4 FoxP3 GranzB | -0.464832836 | 7.56E-07 | 6.83E-05 |
|  | Firmicutes:Ruminococcaceae | CD4 FoxP3 GranzB | -0.245585611 | 0.012404539 | 0.099236316 |
|  | Fusobacteria:Sneathia | CD4 FoxP3 GranzB | -0.454221404 | 1.44E-06 | 0.000108074 |
|  | Proteobacteria:Enterobacter | CD4 FoxP3 GranzB | -0.394652487 | 3.71E-05 | 0.000793945 |
|  | Proteobacteria:Klebsiella | CD4 FoxP3 GranzB | 0.245953955 | 0.012269676 | 0.099236316 |
|  | Proteobacteria:Parasutterella | CD4 FoxP3 GranzB | 0.427878287 | 6.54E-06 | 0.000252731 |
|  | Proteobacteria:Proteus | CD4 FoxP3 GranzB | -0.419151877 | 1.05E-05 | 0.000349849 |
|  | Firmicutes:Catenibacterium | CD4 IL-35 | 0.399881345 | 0.00649708 | 0.059064368 |
|  | Proteobacteria:Proteus | CD4 IL-35 | -0.40958128 | 0.0052066 | 0.048811871 |
|  | Candidate-division-TM7:Candidate-division-TM7 | CD8 CD25 | 0.296033758 | 0.002396543 | 0.024371625 |
|  | Firmicutes:Clostridiales | CD8 CD25 | 0.303030303 | 0.00186209 | 0.01960095 |
|  | Actinobacteria:Atopobium | CD8 CTLA4 | -0.345979068 | 0.000344034 | 0.005432115 |
|  | Actinobacteria:Eggerthella | CD8 CTLA4 | 0.438224938 | 3.66E-06 | 0.000183187 |
|  | Actinobacteria:Gardnerella | CD8 CTLA4 | 0.325466513 | 0.00079469 | 0.009933626 |
|  | Actinobacteria:Mobiluncus | CD8 CTLA4 | 0.398496466 | 3.06E-05 | 0.000697386 |
|  | Bacteroidetes:Barnesiella | CD8 CTLA4 | 0.319434174 | 0.001005612 | 0.012067346 |
|  | Bacteroidetes:Odoribacter | CD8 CTLA4 | 0.398371456 | 3.08E-05 | 0.000697386 |
|  | Bacteroidetes:S24-7 | CD8 CTLA4 | -0.327039676 | 0.000746777 | 0.009533329 |
|  | Candidate-division-TM7:Candidate-division-TM7 | CD8 CTLA4 | -0.426215088 | 7.16E-06 | 0.000252731 |
|  | Firmicutes:Blautia | CD8 CTLA4 | 0.329245854 | 0.00068403 | 0.009327678 |
|  | Firmicutes:Catenibacterium | CD8 CTLA4 | -0.413558364 | 1.41E-05 | 0.000423638 |
|  | Firmicutes:Clostridiales | CD8 CTLA4 | -0.451000372 | 1.75E-06 | 0.000116368 |
|  | Firmicutes:Epulopiscium | CD8 CTLA4 | -0.31172552 | 0.001349118 | 0.014990195 |
|  | Firmicutes:Firmicutes | CD8 CTLA4 | 0.263522383 | 0.007155162 | 0.064076079 |
|  | Firmicutes:Gemella | CD8 CTLA4 | 0.4009587 | 2.70E-05 | 0.000675788 |
|  | Firmicutes:Lactococcus | CD8 CTLA4 | -0.350900047 | 0.000279005 | 0.004524407 |
|  | Firmicutes:Megamonas | CD8 CTLA4 | -0.352686384 | 0.000258354 | 0.004305893 |
|  | Firmicutes:Parvimonas | CD8 CTLA4 | -0.463970902 | 7.97E-07 | 6.83E-05 |
|  | Firmicutes:Ruminococcaceae | CD8 CTLA4 | -0.275048469 | 0.004926245 | 0.046916618 |
|  | Fusobacteria:Sneathia | CD8 CTLA4 | -0.435248587 | 4.34E-06 | 0.000185828 |
|  | Proteobacteria:Enterobacter | CD8 CTLA4 | -0.332378159 | 0.00060321 | 0.008617291 |
|  | Proteobacteria:Klebsiella | CD8 CTLA4 | 0.30933563 | 0.001475486 | 0.016096206 |
|  | Proteobacteria:Parasutterella | CD8 CTLA4 | 0.38761081 | 5.23E-05 | 0.001081938 |
|  | Proteobacteria:Proteus | CD8 CTLA4 | -0.464766712 | 7.59E-07 | 6.83E-05 |
|  | Actinobacteria:Atopobium | CD8 IL-10 | 0.345256548 | 0.00035468 | 0.005456609 |
|  | Actinobacteria:Gardnerella | CD8 IL-10 | -0.38591365 | 5.68E-05 | 0.001135123 |
|  | Actinobacteria:Mobiluncus | CD8 IL-10 | -0.376219138 | 8.98E-05 | 0.001633426 |
|  | Bacteroidetes:Barnesiella | CD8 IL-10 | -0.328111971 | 0.00071565 | 0.009383234 |
|  | Bacteroidetes:Odoribacter | CD8 IL-10 | -0.330774778 | 0.000643417 | 0.008977918 |
|  | Bacteroidetes:S24-7 | CD8 IL-10 | 0.371250264 | 0.000113054 | 0.001995064 |
|  | Candidate-division-TM7:Candidate-division-TM7 | CD8 IL-10 | 0.521746941 | 1.59E-08 | 7.64E-06 |
|  | Firmicutes:Blautia | CD8 IL-10 | -0.258362353 | 0.008414029 | 0.072120251 |
|  | Firmicutes:Catenibacterium | CD8 IL-10 | 0.318651549 | 0.001036432 | 0.012187491 |
|  | Firmicutes:Clostridiales | CD8 IL-10 | 0.494003469 | 1.14E-07 | 1.71E-05 |
|  | Firmicutes:Epulopiscium | CD8 IL-10 | 0.249141621 | 0.011155022 | 0.091970167 |
|  | Firmicutes:Firmicutes | CD8 IL-10 | -0.26130329 | 0.007674548 | 0.066735198 |
|  | Firmicutes:Gemella | CD8 IL-10 | -0.426729874 | 6.96E-06 | 0.000252731 |
|  | Firmicutes:Lactococcus | CD8 IL-10 | 0.31815947 | 0.001056249 | 0.012187491 |
|  | Firmicutes:Megamonas | CD8 IL-10 | 0.302258441 | 0.001915233 | 0.019812756 |
|  | Firmicutes:Parvimonas | CD8 IL-10 | 0.270866525 | 0.005650902 | 0.052162175 |
|  | Firmicutes:Ruminococcaceae | CD8 IL-10 | 0.494549189 | 1.10E-07 | 1.71E-05 |
|  | Fusobacteria:Sneathia | CD8 IL-10 | 0.397989505 | 3.14E-05 | 0.000697386 |
|  | Proteobacteria:Enterobacter | CD8 IL-10 | 0.279732282 | 0.004213913 | 0.040779804 |
|  | Proteobacteria:Klebsiella | CD8 IL-10 | -0.327981203 | 0.000719381 | 0.009383234 |
|  | Proteobacteria:Parasutterella | CD8 IL-10 | -0.261930431 | 0.007524492 | 0.066392575 |
|  | Proteobacteria:Proteus | CD8 IL-10 | 0.439186049 | 3.47E-06 | 0.000183187 |
|  | Firmicutes:Epulopiscium | CD8 IL-35 | -0.467566796 | 0.001201738 | 0.013604584 |
|  | Firmicutes:Epulopiscium | CD8 Lag3 | -0.292090518 | 0.002755339 | 0.027553394 |
|  | Proteobacteria:Parasutterella | CD8 Lag3 | 0.257760796 | 0.008572796 | 0.072446166 |
|  | Actinobacteria:Gardnerella | CD8 PD1 | 0.374802267 | 0.011189704 | 0.091970167 |
|  | Firmicutes:Firmicutes | CD8 PD1 | 0.431809201 | 0.003057817 | 0.030076892 |
| Week 28 | Actinobacteria:Eggerthella | CD4 CD25 | -0.287613802 | 0.004940918 | 0.063062143 |
|  | Actinobacteria:Eggerthella | CD4 FoxP3 | -0.275311833 | 0.007239029 | 0.083269945 |
|  | Actinobacteria:Eggerthella | CD4 FoxP3 CD25 | -0.299357393 | 0.003377906 | 0.049432768 |
|  | Firmicutes:Firmicutes | CD4 GITR | 0.427582851 | 8.48E-05 | 0.001754873 |
|  | Firmicutes:Ruminococcaceae | CD4 IL-35 | 0.321183355 | 0.003900173 | 0.054421025 |
|  | Bacteroidetes:Odoribacter | CD4 PD1 | -0.617202711 | 1.38E-09 | 4.14E-07 |
|  | Fusobacteria:Sneathia | CD4 PD1 | -0.53706703 | 3.35E-07 | 3.35E-05 |
|  | Firmicutes:Epulopiscium | CD4 PD1 | 0.533343341 | 4.18E-07 | 3.58E-05 |
|  | Bacteroidetes:S24-7 | CD4 PD1 | 0.515972036 | 1.13E-06 | 6.81E-05 |
|  | Firmicutes:Catenibacterium | CD4 PD1 | -0.504112415 | 2.17E-06 | 9.32E-05 |
|  | Proteobacteria:Klebsiella | CD4 PD1 | 0.490208146 | 4.52E-06 | 0.000169678 |
|  | Candidate-division-TM7:Candidate-division-TM7 | CD4 PD1 | 0.486132281 | 5.58E-06 | 0.000196767 |
|  | Firmicutes:Lactococcus | CD4 PD1 | 0.471998608 | 1.13E-05 | 0.000365868 |
|  | Actinobacteria:Gardnerella | CD4 PD1 | -0.447014348 | 3.63E-05 | 0.000989886 |
|  | Firmicutes:Parvimonas | CD4 PD1 | -0.439544478 | 5.06E-05 | 0.001226215 |
|  | Actinobacteria:Mobiluncus | CD4 PD1 | -0.439327982 | 5.11E-05 | 0.001226215 |
|  | Firmicutes:Gemella | CD4 PD1 | -0.430281649 | 7.56E-05 | 0.001620374 |
|  | Actinobacteria:Atopobium | CD4 PD1 | -0.42512311 | 9.41E-05 | 0.001881961 |
|  | Proteobacteria:Proteus | CD4 PD1 | -0.416451265 | 0.000134834 | 0.002528138 |
|  | Proteobacteria:Parasutterella | CD4 PD1 | 0.331735566 | 0.002821014 | 0.044542321 |
|  | Firmicutes:Megamonas | CD4 PD1 | 0.330240851 | 0.002955495 | 0.045469146 |
|  | Firmicutes:Blautia | CD4 PD1 | 0.309533955 | 0.005505433 | 0.067413469 |
|  | Firmicutes:Firmicutes | CD4 TGFb | 0.3413649 | 0.000759587 | 0.013810679 |
|  | Firmicutes:Lactococcus | CD4 TGFb | 0.292022194 | 0.004291398 | 0.058519062 |
|  | Actinobacteria:Mobiluncus | CD4 TGFb | -0.287733547 | 0.00492218 | 0.063062143 |
|  | Firmicutes:Blautia | CD4 TGFb | 0.283090588 | 0.005696857 | 0.068362287 |
|  | Candidate-division-TM7:Candidate-division-TM7 | CD4 TGFb | 0.274084526 | 0.007513375 | 0.083481948 |
|  | Firmicutes:Epulopiscium | CD8 CTLA4 | 0.575870019 | 1.26E-09 | 4.14E-07 |
|  | Bacteroidetes:Odoribacter | CD8 CTLA4 | -0.54163184 | 1.73E-08 | 3.46E-06 |
|  | Firmicutes:Lactococcus | CD8 CTLA4 | 0.534687642 | 2.84E-08 | 4.26E-06 |
|  | Fusobacteria:Sneathia | CD8 CTLA4 | -0.501653787 | 2.60E-07 | 3.12E-05 |
|  | Firmicutes:Catenibacterium | CD8 CTLA4 | -0.491229185 | 4.98E-07 | 3.73E-05 |
|  | Actinobacteria:Atopobium | CD8 CTLA4 | -0.487683518 | 6.18E-07 | 4.12E-05 |
|  | Actinobacteria:Gardnerella | CD8 CTLA4 | -0.4744326 | 1.36E-06 | 7.40E-05 |
|  | Firmicutes:Gemella | CD8 CTLA4 | -0.472971185 | 1.48E-06 | 7.40E-05 |
|  | Actinobacteria:Mobiluncus | CD8 CTLA4 | -0.466360947 | 2.16E-06 | 9.32E-05 |
|  | Proteobacteria:Proteus | CD8 CTLA4 | 0.461307721 | 2.88E-06 | 0.000115089 |
|  | Firmicutes:Megamonas | CD8 CTLA4 | 0.435353529 | 1.16E-05 | 0.000365868 |
|  | Proteobacteria:Parasutterella | CD8 CTLA4 | 0.432748392 | 1.32E-05 | 0.000397263 |
|  | Bacteroidetes:S24-7 | CD8 CTLA4 | 0.42561609 | 1.90E-05 | 0.000542427 |
|  | Firmicutes:Parvimonas | CD8 CTLA4 | -0.405387497 | 5.05E-05 | 0.001226215 |
|  | Bacteroidetes:Barnesiella | CD8 CTLA4 | 0.400996703 | 6.20E-05 | 0.001429787 |
|  | Candidate-division-TM7:Candidate-division-TM7 | CD8 CTLA4 | 0.399889736 | 6.52E-05 | 0.00144893 |
|  | Proteobacteria:Enterobacter | CD8 CTLA4 | -0.38537522 | 0.000125249 | 0.002424179 |
|  | Proteobacteria:Klebsiella | CD8 CTLA4 | 0.325322101 | 0.001377063 | 0.023606788 |
|  | Firmicutes:Blautia | CD8 CTLA4 | 0.310682313 | 0.002306231 | 0.038437188 |
|  | Firmicutes:Clostridiales | CD8 CTLA4 | 0.305558667 | 0.002745916 | 0.044528369 |
|  | Actinobacteria:Coriobacteriaceae | CD8 CTLA4 | -0.27478581 | 0.007355512 | 0.083269945 |
|  | Firmicutes:Blautia | CD8 Lag3 | 0.286956113 | 0.005044971 | 0.063062143 |
|  | Proteobacteria:Proteus | CD8 Lag3 | 0.276527827 | 0.00697597 | 0.082070231 |
|  | Bacteroidetes:S24-7 | CD8 PD1 | 0.357478923 | 0.001219495 | 0.021520496 |
|  | Firmicutes:Lactococcus | CD8 PD1 | 0.326025423 | 0.00336618 | 0.049432768 |
|  | Bacteroidetes:Odoribacter | CD8 PD1 | -0.322129643 | 0.003790243 | 0.054146326 |
|  | Firmicutes:Firmicutes | CD8 TGFb | 0.288127573 | 0.004860966 | 0.063062143 |
|  | Firmicutes:Blautia | CD8 TGFb | 0.272446447 | 0.007893811 | 0.086114304 |
| Week 62 | Firmicutes:Epulopiscium | CD8 PD1 | 0.500951325 | 0.000211115 | 0.068021562 |
|  | Candidate-division-TM7:Candidate-division-TM7 | CD8 PD1 | 0.498834964 | 0.000226739 | 0.068021562 |

A

B

**Figure S3. Gating strategy for in vitro treatment of PBMCs with inactivated bacteria**. **Panel A** shows unstimulated PBMCs**. Panel B** shows PBMCs stimulated with UV-inactivated culture products of *Lactococcus lactii* for 7 days**.**

**Figure S4. Ex vivo treatment of HEU PBMCs with inactivated bacteria did not affect the proportions of Treg subsets.** Data were generated using PBMCs from 6 HEUs each treated for 7 days with the UV-inactivated bacterial products indicated on each graph and with medium control. P values were calculated with Wilcoxon matched-pairs signed rank test.

**Figure S5. Ex vivo treatment of adult PBMC with inactivated bacteria reproduces the effect on HUU PBMC.** Data were generated using PBMC from adult platelet donation leukopack aliquots. Each aliquot was treated for 7 days with the UV-inactivated bacterial products indicated on each graph and with medium control. A formal statistical analysis was not performed due to the low number of experiments.
